# The complete chloroplast genome of *Musa beccarii*

**DOI:** 10.1080/23802359.2020.1775513

**Published:** 2020-06-08

**Authors:** Huimin Feng, You Chen, Xiaoxiong Xu, Hongli Luo, Yaoting Wu, Chaozu He

**Affiliations:** a Hainan Key Laboratory for Sustainable Utilization of Tropical Bioresources, Institute of Tropical Agriculture and Forestry, Hainan University, Haikou, China; b Hainan Tropical Ocean University, Sanya, China

**Keywords:** *Musa becccarii*, chloroplast genome, illumina sequencing, phylogenetic analysis

## Abstract

*Musa becccarii* N.W. Simmonds is one of the most important wild banana species native to Borneo. The chromosome number, 2n = 18, is new to the genus *Musa*. Wild populations of *M. beccarii* have been reduced enormously due to massive land clearing for oil palm plantations. In this study, we report the complete chloroplast genome of *Musa beccarii* by next-generation sequencing (NGS). The total length of the complete chloroplast genome was 168,457 bp, and the overall GC content of the whole genome is 36.8%. The cp genome of *Musa beccarii* contained a pair of inverted repeat regions of 34,819 bp, which were separated by the large single copy of 88,166bp and the small single copy of 11,059 bp. It encoded 114 genes, including 79 protein-coding genes, 31 tRNA genes, and 4 rRNA ribosomal genes. The most genes occur as a single copy, while 21 gene species occur in double copies. The phylogenetic analysis demonstrateds *Musa becccarii* formed a single branch among genus *Musa*. This complete chloroplast genome will provide important information for conservation and identification of species of *Musa* spp.

*Musa becccarii* N.W. Simmonds belongs to *Musa*, Musacaea, is an endangered endemic species to Borneo (Häkkinen et al. 2005). The chromosome number of *M. beccarii* is 2n = 18 (Häkkinen et al. 2007), while other wild banana species is 2n = 22 (Sect. *Musa*) or 2n = 20 (Sect. *Callimusa*). It has high ornamental value with the colorful flowers. Wild populations of *M. beccarii* have been reduced enormously due to massive land clearing for oil palm plantations in the eastern part of Sabah (Häkkinen et al. 2007). Only a few *M. beccarii* populations could be found in the wild. The information of chloroplast genomes has been extensively applied in understanding plant genetic diversity and evolution that are of great importance in conservation genetics. The complete chloroplast genome of *M. beccarii* will greatly enhance the conservation efforts of this endangered plant species, which harbors precious gene resources for banana breeding programs in the future.

An individual of *M. beccarii* was collected from Bioversity International’s ITC (ITC1070) and deposited at Hainan Tropical Ocean University (Accession number: M08). Total genomic DNA was extracted from fresh young leaves using modified CTAB method (Doyle and Doyle 1987). Purified total genomic DNA was used for sequencing with Illumina Hiseq X Ten platform. Approximately 6.0 Gb raw reads were obtained, and clean reads (5.5 G) were further assembled into contigs using MITObim v1.8 (Hahn et al. 2013) and SOAPdenovo v2.04 (Luo et al. 2012), with *M. acuminata* subsp. *malaccensis* chloroplast genome sequence (Genebank accession number: HF677508.1) as a reference genome (Martin et al. 2013). The circular map was obtained using the online program Organellar Genome Draw tool v1.2 (Lohse et al. 2013). The annotated chloroplast genomic sequence was submitted to the Genbank with the accession number MK012089.

The complete chloroplast genome of *M. beccarii* was 168,457 bp in length and contained a pair of inverted repeat regions of 34,819 bp, which were separated by the large single copy of 88,166 bp and the small single copy of 11,059 bp. The chloroplast genome contained 114 unique genes, including 79 protein-coding genes, 31 tRNA genes, and 4 rRNA genes. Among these genes, 15 genes (petB, petD, atpF, ndhA, ndhB, rpoC1, rps16, rpl2, rpl16, trnA-Lys, trnG-UCC, trnL-UAA, trnV-UAC, trnI-GAU, trnA-UGC) have one intron and 2 genes (clpP and ycf3) have two introns. Most of the genes occurred as a single copy, but 9 protein-coding genes (ndhB, ndhH, rps7, rps12, rps15, rpl2, rpl23, ycf1, ycf2), 8 tRAN genes (trnI-CAU, trnV-GAC, trnI-GAU, trnA-UGC, trnR-ACG, trnN-GUU, trnL-CAA, trnH-GUG) and 4 rRNA genes (rrn16, rrn23, rrn4.5, rrm5) in the IR regions are duplicated. The overall GC content of *M. beccarii* whole chloroplast genome is 36.8% and the corresponding values in LSC, SSC and IR regions are 35.16%, 31.14% and 39.87%, respectively.

To investigate the phylogenetic relationships with closely related species, a maximum likelihood phylogenetic tree was constructed based on chloroplast genomes of 6 published species of Musaceae with the program IQTREE v1.6 (Nguyen et al. 2015; Hoang et al. 2018). *Heliconia collinsiana* (JX088660) were used as the outgroup for tree rooting. The result reveals that of *M. beccarii* formed a single branch among genus *Musa*. The complete chloroplast genome of *M. beccarii* and defined its phylogenetic would provide valuable information for the genetic evaluation, population genetic study and conservation of the endangered species (Figure 1).

## Data Availability

The data that support the findings of this study are openly available in national center for biotechnology information (NCBI) at https://www.ncbi.nlm.nih.gov/, reference number [MK012089]. Maximum likelihood tree based on the complete chloroplast genome sequences of 6 Musacaea species. The numbers on the branches are bootstrap values. GenBank accession numbers are shown in the figure.
